# Piloting a Patient-Driven Online Survey to Better Understand Cancer in Adolescents and Young Adults (AYA) in the United States

**DOI:** 10.7759/cureus.63749

**Published:** 2024-07-03

**Authors:** Lynda Beaupin, Scott Borinstein, Nicholas D Yeager, Denise Rokitka, Jennifer Schweitzer, Odochi Uwazurike, John Senall, Earnest Amankwah, Peter H Shaw

**Affiliations:** 1 Cancer and Blood Disorders Institute, Johns Hopkins All Children's Hospital, St. Petersburg, USA; 2 Pediatric Hematology/Oncology, Vanderbilt Children’s Hospital/Vanderbilt University School of Medicine, Nashville, USA; 3 Pediatric Oncology, Nationwide Children’s Hospital, Columbus, USA; 4 Department of Pediatrics, Roswell Park Comprehensive Cancer Center, Buffalo, USA; 5 Medical Education, Mobile First Media, LLC, Buffalo, USA; 6 Clinical &amp; Translational Science Institute/Data Science, Medical College of Wisconsin, Milwaukee, USA; 7 Pediatrics/Oncology, Children's Wisconsin/Medical College of Wisconsin, Milwaukee, USA

**Keywords:** access to health care, cancer survivorship, registry, cancer, adolescent and young adult

## Abstract

Adolescents and young adults (AYAs) with cancer are a unique patient population in oncology. An opt-in, secure online survey was conducted among a general population of AYA patients and survivors to better understand the current landscape of AYA cancer. A 28-item online survey was designed for cancer patients and survivors diagnosed between the ages of 18 and 39 years. It comprised questions about demographics, treatment site, clinical trial involvement, support services available, and impact on employment, schooling, and finances. A total of 590 patients registered and 447 (76%) completed the survey. This online exercise was found to be feasible and can serve as an effective method to survey the AYA cancer population.

## Introduction

Adolescents and young adult (AYA) cancer patients aged 15-39 years had demonstrated inferior survival rates for many malignancies compared to older and younger patients from the 1970s through 2000 [1], albeit some progress in survival rates over the last 10 years [2]. The reasons for this are multifactorial, including a low index of suspicion for cancer in the AYA population, delay in diagnosis, lack of insurance, referral patterns of these patients, lower rates of clinical trial enrollment, poor treatment compliance, and poor understanding of the biology of AYA malignancies [3]. Although pediatric cancer accounts for <1% of all new cases annually in the United States, AYA patients make up 4.6% of all new cases, which equates to more than 88,000 new cancer patients per year, and account for 1.5% of all cancer deaths, or approximately 9,000 per year [4]. Our understanding of cancer in this unique population was mostly derived from retrospective epidemiological studies, and there is a lack of research based on self-reported data. While most pediatric patients are treated at academic medical centers where clinical trial enrollment is high, most young adults >18 years are treated at community hospitals [5,6], where they are significantly less likely to be enrolled in therapeutic or non-therapeutic clinical trials, leading to a knowledge gap related to this vulnerable patient population [7]. 

In 2005, the National Cancer Institute (NCI), with support from the Lance Armstrong Foundation (LIVESTRONG), convened a Progress Review Group on AYA cancer [8]. Its recommendations led to the first national cohort study of AYA cancer patients in the United States: the Adolescent and Young Adult Health Outcomes and Patient Experience (AYA HOPE). Study participants diagnosed between the ages of 15 and 39 years were identified from seven NCI Surveillance, Epidemiology, and End Results (SEER) cancer registries, and 524 participants who were diagnosed between July 1, 2007, and October 31, 2008, with common AYA cancers were enrolled [9]. Researchers in Germany performed the AYA-Leipzig study, a longitudinal study that looked into different aspects of the lives of AYA cancer survivors and elucidated various needs of these patients regarding psychological distress and quality of life. Most of their study subjects were recruited through rehabilitation clinics and acute care hospitals [10-13]. Also, recently two multicenter studies examined the financial and employment-related impact among AYA cancer survivors, one involving only women [14] and the other both men and women [15].

Although AYA HOPE has provided comprehensive information on AYAs, a simplified approach to assess the current landscape of AYA survivors may be an effective method to engage with and learn more about this unique population. The Consortium of Adolescent and Young Adult Cancer Centers (CAYACC) was established to improve our understanding of AYA cancer in the United States. An online, web-based platform was created to collect self-reported health and psychosocial information from AYAs both in academic and community settings. The goal of this study was to assess the feasibility of an opt-in, secure online survey to collect data from a broader landscape of AYA patients and survivors.

## Materials and methods

In this cross-sectional study, an internet-based online survey was designed for cancer patients and survivors diagnosed between the ages of 18 and 39 years to collect information about their cancer diagnosis, treatment setting, clinical trial access and enrollment, insurance status, social support, and fertility preservation utilization. The inclusion criteria were as follows: individuals diagnosed with cancer between the ages of 18 and 39 years. Respondents could be older than 39 years at the time of survey completion if they had been diagnosed and treated within the established age range. The survey was open from April 2018 to February 2019. Patients were recruited through social media and outreach to AYA cancer programs and support organizations. Patients self-enrolled and completed a 28-question confidential study. No identifying data were collected. Only completed survey data were included for analysis. Survey participation implied informed consent. The survey was approved by the Roswell Park Comprehensive Cancer Center (RPCCC)’s Institutional Review Board. Data were compiled through a website and securely stored on a REDCap database through the RPCCC [16].

Dichotomous items involved questions about insurance at the time of diagnosis, lapses in insurance during treatment, treatment status, awareness of clinical trials, and physician’s recommendation for participation in a clinical trial. Data on the duration of symptoms, treatment setting, selection of a provider, quality of care, and information on side effects were ascertained using the questions indicated in the survey. Survey responses were summarized as counts and percentages and age at diagnosis was summarized as median and range. We did not test any hypothesis in this study and hence no p-values are presented (see the Appendices for the full questionnaire).

## Results

Demographics

A total of 590 patients registered and 447 (76%) completed the survey, as summarized in Table 1. The median age of respondents was 33 years at the time of survey entry (range: 18-60 years) with an IQR of 11. Most patients were female (n = 380, 85%) and self-described as Caucasian (n = 420, 94%). Most patients (n = 281, 63%) were married or living with a partner and nearly half (n = 210, 47%) were parents. Most (n = 360, 80%) had a college degree.

**Table 1:** Characteristics of the participants (N = 447)

Question		
Age in years, median (range)	33 (18-60)	
Sex	N	%
Female	380	85%
Male	67	15%
Race (can select more than one)		
White	420	94%
Black/African American	13	3%
Hispanic and Latino	18	4%
Asian	17	4%
Native American/Alaska Native	5	1%
Personal/family situation at the time of the survey		
Married/living with partner	281	63%
Single and never married	172	38%
Divorced or separated	24	5%
Raising children at the time of the survey		
Yes	210	47%
No	237	53%
Education completed		
Some or completed high school	25	6%
Some college/vocational school	62	14%
College degree/postgraduate degree	360	80%
Health insurance at diagnosis		
Yes	421	94%
No	24	5%
I don’t know	2	0.40%
Lapses in health insurance during treatment		
Yes	51	11%
No	390	87%
I don’t know	6	1%

Diagnosis and access to care

**Figure 1:**
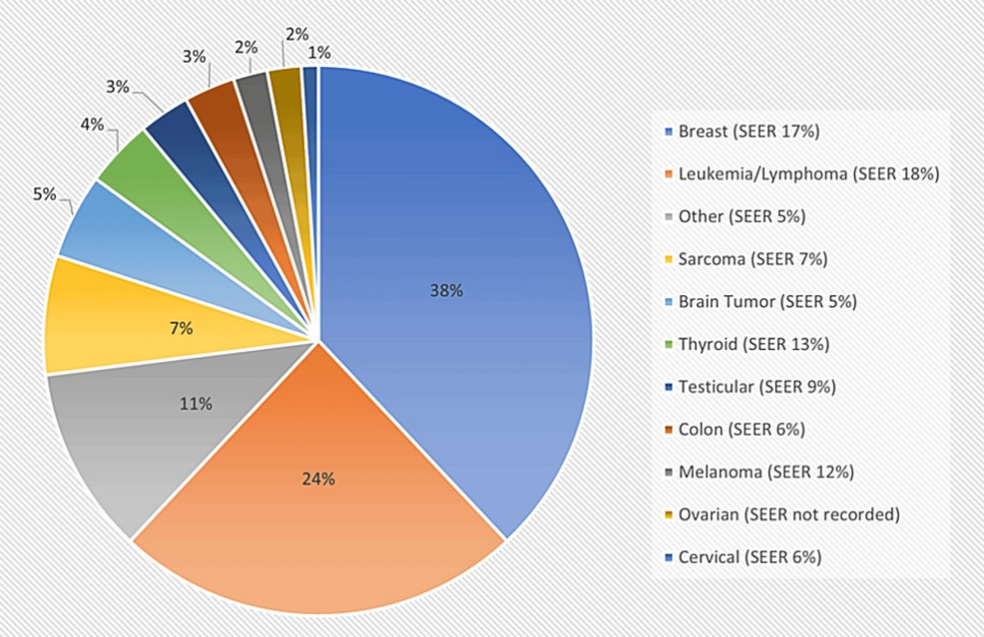
Distribution of cancer diagnoses compared to SEER data SEER: Surveillance, Epidemiology, and End Results

The distribution of cancer diagnoses among the participants is shown in Figure 1, with the distribution from the SEER data included for comparison. The most common diagnoses were breast cancer, which comprised more than one-third (N = 170, 38%) of all survey responses, and leukemia and lymphoma (N = 107, 24%).

Table 2 summarizes the characteristics of the patient cancer experience. Most patients experienced symptoms for weeks to months before seeking medical care (n = 265, 63%). Only a small proportion (n = 45, 11%) stated that their symptoms lasted more than one year before physician evaluation, and over one quarter (n = 137, 32%) had either no symptoms or symptoms for days before medical care. Most received or were receiving care at a cancer center or children’s hospital (n = 326, 73%) rather than at a community hospital or private practice. Nearly all patients were satisfied with the cancer treatment they received, most rating it as “very good” or “excellent” (n = 368, 82%). When asked if clinical trials were available at diagnosis, 50% were not sure (n = 223), 28% stated yes (n = 126), and 22% no (n = 98). When asked about overall clinical trial participation, 64% of patients (n = 286) stated that they had participated, 31% had not (n = 139), and 5% did not know (n = 22). Many participants were actively receiving therapy at the time of completion of the survey (n = 183, 41%).

**Table 2:** Characteristics of cancer experience (N = 447)

Question	N	%
How long were you experiencing symptoms before seeking care?		
Weeks	134	32%
Months	131	31%
Days	73	17%
No symptoms – found on a routine visit	64	15%
One year or longer	45	11%
Treatment setting		
Cancer center	277	62%
Community hospital or community clinic	58	13%
Pediatric cancer center or children's hospital	49	11%
Independent adult cancer oncology practice	49	11%
Other	14	3%
How did you choose your primary oncology provider? (Can select more than one)		
My doctor referred me	287	64%
I did my own research	176	39%
Shortest distance from home	100	22%
Based on my insurance	81	18%
Currently receiving cancer treatment		
Yes	183	41%
No	264	59%
Aware of available clinical trials		
Yes	126	28%
No	98	22%
I don’t know	223	50%
Did your doctor recommend a clinical trial?		
Yes	114	25%
No	316	71%
I don’t know	17	4%
Quality of care you received		
Excellent	210	47%
Very good	158	35%
Good	53	12%
Fair	23	5%
Poor	3	0.70%
Information on long-term side effects		
I have enough information	246	55%
I need some more information	124	28%
I need much more information	37	8%
Does not apply	40	9%

Education and employment

Most patients (n = 402, 90%) were engaged in full-time employment or school. Nearly all patients needed to make significant work/educational changes due to their cancer diagnosis, such as needing to take extended time off (n = 191, 43%), switching to part-time (n = 48, 10%), or stopping work/school completely (n = 114, 26%). Only 23% (n = 103) of the patients reported that cancer had no impact on either work or school. Of note, 45% of the patients (n = 201) in school reported no impact on their education plans, while 21% (n = 94) had a somewhat negative impact, 7% a very negative impact (n = 31), 6% a somewhat positive impact (n = 27), and 6% a very positive impact (n = 27). Regarding the effect of cancer on work plans, 42% reported a somewhat negative impact (n = 188), 24% no impact (n = 107), 19% a very negative impact (n = 85), 8% a very positive impact (n = 36), and 5% a somewhat positive impact (n = 22).

Financial impact and social support

Most (79%) participants stated that cancer had a negative impact financially (n = 353). A small segment (15%) stated that the cancer diagnosis had no impact (n = 67) and only a few (4%) thought that it had a positive financial impact (n = 18). One quarter (27%) received professional advice to help figure out payment of healthcare costs (n = 121). Psychosocial support was provided mostly by family (77% mother, 54% father, 36% sister, 22% brother) in addition to friends (60%) and a significant other (12%). Very few (2%) reported no support (n = 9). Many patients relied on psychosocial support groups, with 39% participating in person (n = 174) and 62% in an online or social media support group (n = 277).

Knowledge of long-term side effects and fertility risks

Regarding knowledge about the long-term side effects of therapy, nearly half (n = 197, 44%) felt that they were adequately educated. Approximately one-third (n = 156, 35%) needed some additional information and 18% (n = 80) needed much more information. When asked specifically about the fertility risks of their therapy, 46% (n = 206) thought they had been adequately informed, 22% (n = 98) needed some more information and 10% (n = 45) needed much more information. When asked if someone at their treatment center discussed the potential impact of therapy on fertility, 73% (n = 326) said yes, 22% (n = 98) no, and 4% (n = 18) were unsure. When asked if they had been offered fertility preservation services (sperm banking oocyte/embryo cryopreservation), 64% (n = 287) stated they did not use any of these services, 14% (n = 63) banked sperm, 14% (n = 63) froze oocytes, and 7% (n = 31) froze embryos. The cost was a major barrier for nearly half (n = 215, 48%) of these patients, and two-thirds (n = 291, 65%) stated that at the time of diagnosis, fertility preservation was not a priority to them, despite it hurting future family planning for 62% (n = 277).

Insurance coverage

Nearly all patients (n = 420, 94%) had insurance at diagnosis; however, 12% (n = 54) reported that they had lapses in insurance coverage during or after treatment; 17% (n = 76) of patients stated that their decision about where to receive cancer care was dictated by insurance coverage. Most patients (n = 340, 76%) had private health insurance through an employer or school. A small segment (n = 49, 11%) were covered through their parents’ insurance plan. A few respondents (n = 54, 12%) were enrolled in Medicaid, while 5% (n = 22) were insured through a federal or state exchange through the Affordable Care Act (ACA). When asked the question “Were there any tests or treatments your oncologist recommended for cancer not covered by insurance?”, 35% (n = 156) responded “yes.” In instances where the tests and treatments were not covered, most patients (n = 344, 77%) stated that they still received them.

## Discussion

AYA cancer patients face unique challenges that hinder their ability to receive optimal health care. The models of cancer care for these patients vary between medical centers and regions, and each medical team has its own unique environment and inherent strengths and challenges in terms of delivering care to its patients. Clinicians who focus on improving AYA cancer outcomes have learned that collaboration and data sharing are paramount in these efforts. It is through cooperative meetings that the idea for CAYACC and this patient-reported survey emerged. 

By engaging in this effort, we were able to collect meaningful data describing the AYA cancer care climate effectively and economically from around the country in under one year, with 590 unique patients starting and 447 completing the full survey (76%). The CAYACC registry was created as a user-friendly platform and registered a substantial group of patients in a short period, demonstrating that such a tool is feasible and can be useful in studying this population.

Our study has certain limitations. We acknowledge that updated AYA SEER data from 2014-2018 exists [4] and is now available; however, the data is less granular and shows similar disease numbers compared to earlier SEER data, prompting us to choose to compare our results to the 2000-2009 data [17]. While we were able to enroll many AYA patients and survivors of cancer, most of our respondents were female, white, well-insured, and well-educated, likely because a greater percentage of respondents had breast cancer when compared to the SEER data (38% vs 17%). Unfortunately, this demographic distribution limits our ability to adequately assess the cancer experience of patients from lower socioeconomic strata or persons of color. Most of the respondents were treated at a cancer center or pediatric hospital vs. a community hospital. Patients were almost uniformly very happy with the care they received. The quality of care in our survey was subjective, and hence it is unclear whether the high overall satisfaction with the care these patients received (82% reporting “very good” or “excellent”) has any correlation with the setting in which they were treated.

Another shortcoming of our research is that 94% of surveyed patients had insurance at the time of diagnosis, which is most likely skewed to a more advantaged socioeconomic demographic, compared to the majority of AYA cancer patients, who have traditionally been the least insured age group in the United States [18]. The insurance coverage landscape has changed for this population after the passage of ACA in 2010, which allows young adults to remain as dependents until 26 years of age [19]. It is unclear, however, how much of an impact the ACA has had on the insurance coverage of our patients based on the questions in our survey. The AYAs we surveyed continue to report several ongoing challenges, especially financial issues and a lack of clinical trial-related knowledge despite national efforts to improve education and access. Challenges in medical and social areas carry over into survivorship, as our survey reveals.

## Conclusions

Overall, we have demonstrated the feasibility of a patient-driven survey to accelerate the tracking of AYA cancer populations and develop robust databases. There has not been a follow-up to the AYA HOPE study or any other substantial AYA online American survey study to date. Our study, despite its limitations, demonstrates that an online patient-driven registry is feasible, not geographically limited, and treatment center-agnostic while being inexpensive to execute. It has established a method to engage with AYAs, which can be utilized to study this unique population further with more focused, in-depth questionnaires. The scope and reach of future surveys could be further expanded through more aggressive marketing and by increasing the number of cooperating centers to obtain a more representative AYA population in the United States.

## Appendices

**Table 3:** Patient questionnaire

Age
Sex
Female
Male
Other
Race (can select more than one answer)
White
Black/African American
Hispanic and Latino
Asian
Native American/Alaska Native
Native Hawaiian/Other Pacific Islander
Other
Education completed
Some high school
Completed high school
Some college/vocational/training school
Associate degree
College graduate
Postgraduate degree
How long were you experiencing symptoms before seeking care?
1 year or longer
Months
Weeks
Days
No symptoms – found on a routine visit
Treatment setting
Community hospital or community clinic
Pediatric cancer center or children's hospital
Independent adult cancer oncology practice
A cancer center
Other
How did you choose your primary oncology provider? (Can select more than one answer)
My doctor referred me
I did my own research
Shortest distance from home
Insurance status influenced my decision
Currently receiving cancer treatment
Yes
No
Were you aware of available clinical trials?
Yes
No
I don’t know
Have you participated in a clinic trial?
Yes
No
I don’t know
Marital status
Single (never married)
Married or living as married w/partner
Divorced
Separated
Widowed
Currently raising children?
Yes
No
Quality of care
Poor
Fair
Good
Very good
Excellent
Health insurance at diagnosis?
Yes
No
I don’t know
What was the source of your insurance?
Through my or my partner’s employer/school
My parents
Medicaid
Federal/state exchange
None
Did you have a lapse in health insurance during treatment?
Yes
No
I don’t know
Were any tests or treatments your doctor recommended not covered by insurance?
Yes
No
If the test/treatment was not covered, did you receive them anyway?
Yes
No
Do you have knowledge of the long-term side effects of your cancer treatment?
I have enough information
I need some more information
I need much more information
Does not apply
Do you have knowledge about the fertility risks of your cancer treatment?
I have enough information
I need some more information
I need much more information
Does not apply
Did someone at your treatment center discuss the potential impact of therapy on your fertility?
Yes
No
Not Sure
If offered sperm banking/ova/embryo freezing, did you use these services?
No
I banked sperm
I froze ova
I froze embryos
Not sure
The cost of sperm/ova/embryo freezing was too high for me
Agree
Disagree
Preserving my fertility was not a priority for me at the time of diagnosis
Agree
Disagree
Did cancer have an impact on your plans for a family?
A very negative impact
Somewhat negative impact
No Impact
Somewhat positive impact
Very positive impact
What was your school/employment status at the time of diagnosis?
Working full-time
Working part-time
Student full-time
Student part-time
Homemaker/caregiver full-time
Unemployed
What was the immediate impact of your cancer diagnosis on your school/employment?
Took more than 2 weeks off from work
Took more than 2 weeks off from school
Stopped work completely
Stopped school completely
Shifted to part-time work
No impact on work/school
What was the long-term impact of cancer on your education plans?
Very negative impact
Somewhat negative impact
No impact
Somewhat positive impact
Very positive impact
Does not apply
If applicable, what was the long-term impact of cancer on your work plans?
Very negative impact
Somewhat negative impact
No impact
Somewhat positive impact
Very positive impact
Does not apply
Was there a financial impact of your cancer treatment?
Very negative impact
Somewhat negative impact
No impact
Somewhat positive impact
Very positive impact
Does not apply
Did you receive professional advice to help figure out the payment of your medical bills?
Yes
No
Who provided medical care/emotional support? (Can select more than one answer)
Mother
Father
Friends
Sister
Brother
Boyfriend/girlfriend
Spouse
Stepfather
Stepmother
No one
Did you participate in an in-person support group?
Yes
No
Did you participate in an online or social media support group?
Yes
No
Did you ever see a mental health professional in person?
Yes
No
Did you ever see a mental health professional virtually or by phone?
Yes
No
Check all the ways you prefer to receive communication and information about services available to you in your area
Email
Facebook
Postal mail (snail mail)
Instagram
Twitter
Pinterest
